# COVID-19 over the last 3 years in China, what we've learned

**DOI:** 10.3389/fpubh.2023.1209343

**Published:** 2023-07-13

**Authors:** Jiang Shi, Fenghua Chen, Shugong Chen, Haoqing Ling

**Affiliations:** Family Medicine Zhangjiang Community Health Centre, Shanghai, China

**Keywords:** SARS-CoV-2, COVID-19, zero-tolerance, herd immunity, quarantine actions

## Introduction

As of 15 May 2023, the COVID-19 pandemic has resulted in a total of 765,903,278 cases and 6,927,378 cumulative deaths worldwide (1). The novel coronavirus-19 (COVID-19) is caused by an infection of the SARS-CoV-2 virus and was first identified in Wuhan, China in December 2019 (2). Despite the pandemic no longer being the main concern, it is important to review the handling approaches and responses to the viral attack as it may offer insights to readers in facing unprepared future situations.

## Emerging of SARS-CoV-2 virus from unknown to known

At the beginning of December 2019, the severity and seriousness of COVID-19 were overlooked due to a lack of experience by the authorities. The local CDC was unable to provide adequate advice to deal with such a lethal virus, and Wuhan authorities even encouraged residents to participate in functions organized by the local authority to celebrate the Chinese Spring Festival, which resulted in a large outbreak of COVID-19 (3). Following the outbreak, it was recognized by the Chinese CDC experts that the SARS-CoV-2 virus was transmissible between humans by 25 January 2020.

To control and minimize viral transmission, Wuhan authorities declared a state of emergency and implemented a complete lockdown starting on January 23, 2020. However, it was too late as half of the regular residents had already moved to other parts of China, and the COVID-19 virus was transmitted to each province and region of China (4).

## Zero-tolerance policy

To minimize the spread of the SARS-CoV-2 virus, a zero-tolerance policy was implemented, including mandatory mask-wearing, social distancing, and restricted isolation of infected individuals in Fangcang hospitals. The restrictive lockdown of Wuhan city lasted for almost 3 months, during which period nobody was allowed to leave their residences. While this measure proved effective in controlling the outbreak, unintended consequences such as difficulty in obtaining groceries and routine medications during the restricted lockdown, as well as increased psychological and psychiatric problems from pre-existing and/or new patients, were raised.

The lesson learnt is that restrictive quarantine is mandatory in response to SARS-CoV-2 viral transmission, despite the consequential cost at certain levels. However, it is also debatable how long the time period and how restricted the quarantine should be. The original hypothesis of zero-tolerance was based on the belief that if people were away from the viral attack, the virus would be eliminated by itself. Although the lockdown has proven to be effective and necessary in controlling the pandemic, viral mutations are still ongoing without effective vaccination. Nevertheless, the zero-tolerance policy helps protect public health, especially for those who cannot get vaccinated or are immunocompromised.

## Reverse viral transmission to China

Prior to the complete lockdown in China, there were instances of viral transmission to other areas and countries, resulting in outbreaks of COVID-19 in countries such as Italy, the USA, Iran, and Russia (4). Local authorities in China then faced the challenge of handling the reversal of SARS-CoV-2 transmission from other countries (5).

To address this challenge, quarantine measures were immediately implemented at all ports of entry in China for travelers coming from high-risk regions/countries. These measures included up to 3 weeks of isolation in designated quarantine hotels, followed by 1 week of home quarantine. While this measure appeared effective in limiting the transmission of the virus, ongoing debates exist regarding the length and scope of quarantine measures. It is also debatable whether unilateral isolation/quarantine of anyone from abroad is effective in controlling the spread of SARS-CoV-2, as isolation/quarantine without effective vaccination is not the best defense against viral transmission.

Nevertheless, the importance of a quarantine system in controlling viral transmission cannot be overstated. However, concerns have been raised about the resources required to maintain such measures, including food and manpower.

## Co-incidence with flood

It has been reported that SARS-CoV-2 viral mutations have played a critical role in the new waves of COVID-19 pandemic (6). Since its initial emergence, the SARS-CoV-2 virus has mutated from the alpha and beta variants to the highly contagious delta variant, which was first identified in South Africa and has since spread worldwide. The delta variant has caused new waves of infections in many countries (6). In China, the delta strain of the SARS-CoV-2 virus was accidentally leaked by airport cleaners in Jiangsu Province and quickly spread to the surrounding regions, causing chaos. Additionally, a devastating flood occurred in Zhengzhou, the capital of Henan Province, during the outbreak of the delta strain, catching people by surprise and resulting in unacceptably high casualties.

The lesson learned from this incident is that immediate attention must be focused on addressing the immediate disaster at hand, such as moving everyone to a safe location during a flood, regardless of COVID-19 status. Of course, necessary precautions should be taken during the transportation of flood victims (7). Once the immediate situation is resolved, attention should then be paid to SARS-CoV-2 viral transmission (7). Although the wave of delta viral outbreak was eventually brought under control, the lesson learned is that it is necessary to prepare for unexpected disasters with effective alternative plans that should be put in place at the emergence stage.

## Omicron viral infection—Is it the ending of COVID-19?

In early 2022, the omicron strain of SARS-CoV-2 was reported, which caused relatively high morbidity, although mortality remained low (8). The omicron strain was highly transmissible, reaching every country within 4 weeks (8). Individuals with chronic conditions who were unable to receive vaccinations were the most vulnerable to omicron. In response to this strain, different countries adopted different approaches. Western societies offered updated vaccinations to the general population to develop herd immunity, while Chinese authorities implemented one of the strictest lockdowns, particularly in Shanghai. It should be noted that the regular population in Shanghai is around 25 million, with four million individuals transiting daily.

Several proposals were suggested, such as isolating people with natural barriers like the Huangpu river, which separates Shanghai into two halves, or segmenting the city into several pieces. However, a complete and highly restricted lockdown was implemented, where a mandatory negative PCR test for COVID-19 was required within 24 h prior to admission to any hospital or clinic, even in emergency cases such as asthmatic attacks or dialysis patients. This posed significant challenges for medical practitioners in properly handling patients. The lesson learned from this situation is that achieving herd immunity can be more heavily based on effective vaccinations, in addition to allowing a moderate level of viral transmission with controlled levels, such as a relatively low viral load and less virulent strains.

Subsequently, it was realized that strict quarantine measures may not be effective without the availability of effective vaccinations to develop immunity. As a result, the authorities in China completely abolished the 72-h viral negative policy and encouraged people to engage in a fast transitional period in early December 2022, and downgrade the COVID-19 into second dangerous pathogen (9), regardless of their viral status. Admittedly, the COVID-19 pandemic came under control within 2 months of this approach in China (9), despite a rather large mortality and morbidity during the transitional period (10). There has been ongoing debate about which approach is better in defending against the most ferocious virus over recent decades, i.e., herd immunity vs. zero-tolerance.

## Discussion

We have faced the most dangerous virus, SARS-CoV-2, which is both lethal and highly transmissible, with high mortality and morbidity. COVID-19 has had a great impact on the healthcare system, causing the biggest and longest pandemic in over a century and presenting a real challenge to the healthcare systems around the world, regardless of socioeconomic status. It seems that the approaches to be taken into consideration are important, i.e., herd immunity vs. zero-tolerance. More importantly, both herd immunity and zero tolerance are based on effective vaccination and/or physical infection with the SARS-CoV-2 virus. However, the responses to control transmission vary due to different philosophies and understandings of the virus.

Zero-tolerance alone is not sustainable over long periods of time and is not able to offer the general population sufficient immunity to defend against the viral attack, regardless of the period and/or restrictive quarantine. Thus, effective vaccination and/or a fast-track approach to facing the real world, as adopted by the Chinese authorities, is workable, as it has been demonstrated that the outbreaks subsided within 2 months. On the other hand, many countries adopted a gradual and slow easing of restrictions, allowing the general population to develop herd immunity, which was well demonstrated during the World Cup soccer event.

Overall, the COVID-19 pandemic is one of the most dangerous challenges to humans over this century, yet it also offers the biggest opportunity for us to exercise facing such a ferocious attack for any unprepared future. Fortunately, it has been officially declared COVID-19 is now an established and ongoing health issue which no longer constitutes a public health emergency of international concern (PHEIC) (11). It is fundamentally important that we learn from this devastating pandemic to develop effective strategies. Quarantine with effective vaccination would be an ideal way of handling SARS-CoV-2 prior to its mutation.

## Author contributions

JS, HL, FC, and SC designed and wrote the manuscript. All authors contributed to the article and approved the submitted version.
